# *biotextgraph*: graphical summarization of functional similarities from textual information

**DOI:** 10.1093/bioinformatics/btae357

**Published:** 2024-06-08

**Authors:** Noriaki Sato, Yao-zhong Zhang, Zuguang Gu, Seiya Imoto

**Affiliations:** Division of Health Medical Intelligence, Human Genome Center, The Institute of Medical Science, The University of Tokyo, Tokyo 108-8639, Japan; Division of Health Medical Intelligence, Human Genome Center, The Institute of Medical Science, The University of Tokyo, Tokyo 108-8639, Japan; Molecular Precision Oncology Program, National Center for Tumor Diseases (NCT), Heidelberg 69120, Germany; Division of Health Medical Intelligence, Human Genome Center, The Institute of Medical Science, The University of Tokyo, Tokyo 108-8639, Japan

## Abstract

**Summary:**

Functional interpretation of biological entities such as differentially expressed genes is one of the fundamental analyses in bioinformatics. The task can be addressed by using biological pathway databases with enrichment analysis (EA). However, textual description of biological entities in public databases is less explored and integrated in existing tools and it has a potential to reveal new mechanisms. Here, we present a new R package *biotextgraph* for graphical summarization of omics’ textual description data which enables assessment of functional similarities of the lists of biological entities. We illustrate application examples of annotating gene identifiers in addition to EA. The results suggest that the visualization based on words and inspection of biological entities with text can reveal a set of biologically meaningful terms that could not be obtained by using biological pathway databases alone. The results suggest the usefulness of the package in the routine analysis of omics-related data. The package also offers a web-based application for convenient querying.

**Availability and implementation:**

The package, documentation, and web server are available at: https://github.com/noriakis/biotextgraph.

## 1 Introduction

When analyzing biological data, lists of biological entities are often obtained such as genes, transcripts, or bacterial taxonomy identifiers. As a next step, obtaining common biological functions that are significantly associated with the list is important for data interpretation. For instance, when investigating gene expression datasets, lists of gene identifiers are obtained through differential expression or clustering analysis. Investigating genes manually is time-consuming and even impossible for a large number of identifiers. Thus, enrichment analysis (EA) is performed using curated biological databases to summarize common biological functions affected in the gene list instead. As another example of omics data, when differentially abundant taxa and corresponding genes are obtained in microbiome analysis, EA is also applied to determine their importance under biological context (Wu *et al.* 2021). Occasionally, EA generates very few significant terms which makes interpretation difficult. Then, directly examining textual descriptions of the corresponding identifiers aids in the understanding of the functionality and similarity of biological entities.

There are several packages that have been reported for summarizing, analyzing, and visualizing biological textual descriptions for such purposes. *Genes2WordCloud* fetches textual description of genes and makes visual representation using wordclouds, and *GOsummaries* summarizes text obtained through gene set EA by wordclouds and compares them between gene clusters (Baroukh *et al.* 2011, Kolde and Vilo 2015). In addition, *pubmed.mineR* (Rani *et al.* 2015) is an R package that classifies, fetches, and summarizes the articles available in PubMed and the recent package *vissE* visualizes textual information from gene set EA (Bhuva 2024). These packages greatly help to comprehend biological entities from a textual perspective.

Extending to these packages, extracting the biologically relevant set of words inside text is needed to understand the biological functions. In this context, network analysis can help understand the relationships between words and identify important sets of words through network representations of words in text analysis. Co-occurrence network, or correlation network of words can be constructed from textual information and has the capability of concise visualization and intuitive comprehension of a large amount of biomedical text data. This leads to understanding of the functional implications of biological entities. Although there are researches that perform network analysis based on biomedical literature (Jurca *et al.* 2016, Yang *et al.* 2022), and R packages such as *pubmed.mineR* which perform co-occurrence network analysis on PubMed literature, a package is still missing which can conveniently perform network analysis with fetching biomedical textual information from multiple databases across various biological entities in previous research.

Thus, we developed an R package *biotextgraph* for textual analysis of biological entities by graphical summarization. It fetches textual information for biological entities such as genes and bacterial taxa from public databases, and provides functions to analyze and visualize the text mining results. It supports identifying the important set of words and prioritizing the biological entities based on textual information, and comparing them through network analysis. The package can be used with the other packages investigating high-throughput data and aid in integrating text analysis and textual information into routine omics-related analysis and thus contribute to a better understanding of the functions of biological entities. We have applied the package to transcriptome analyses and showed that it can extract sets of biologically meaningful terms that could not be obtained by using biological pathway databases alone. In addition, functional interpretation was possible by directly utilizing the bacterial taxonomy identifiers for querying textual descriptions in microbiome analysis.

## 2 Implementation

### 2.1 Network construction and identification of important words and entities

The *biotextgraph* package constructs a co-occurrence or correlation network of words in texts from various data sources. The intended input is a list of identifiers obtained from the analysis such as differential expression analysis or clustering analysis. First, queried entities are converted for the search in biomedical databases and datasets mentioned in the following sections, and the texts corresponding to the entity list are fetched from the sources. The texts are segmented into words and are subsequently filtered. For concise and informative visualization, prefiltering of words is necessary to avoid listing words not related to functionality. Pre-computed word frequency or text frequency-inverse document frequency (TF-IDF) of words calculated from all texts listed in the databases such as RefSeq summary data are available with the package. The users can choose a threshold for frequency-based, TF-IDF-based, and the other filtering option as well as their combinations to filter the words. A term-document matrix is generated using indicators such as word frequency or TF-IDF of words. Subsequently, correlation or co-occurrence networks are calculated from the matrix. Correlation here refers to the user-defined correlation value between terms in a term-document matrix, while cooccurrence is defined as the number of times terms appear together within the same sentence. The correlation or co-occurrence metrics below a user-defined threshold are set to zero. The network calculation and visualization use R libraries including *igraph* and *ggraph* (Csárdi and Nepusz 2006, Pedersen 2021).

Using the network representation, one can tag a set of words based on hierarchical clustering of words with statistical significance using bootstrapping by *pvclust* (Suzuki and Shimodaira 2006). In addition, after the network is calculated, various graph-based node clustering functions implemented in *igraph* can be applied to class objects generated by the package. This is useful for identifying important sets of words within identifier lists.

As the package focuses on biological entities, the functions for the identification of important identifiers using the network representation of the words are available. The identifiers are scored based on how frequently the important words such as high frequent words occurred within the description of an identifier and can be visualized in the plot. This helps prioritize the identifiers within the list in terms of textual information.

Multiple inferred networks with tagged words or biological entities obtained from multiple sources as above can be combined and compared by taking a union or an intersection of them. This helps to assess the similarity of the relationships of words across sources.

**Figure 1.**
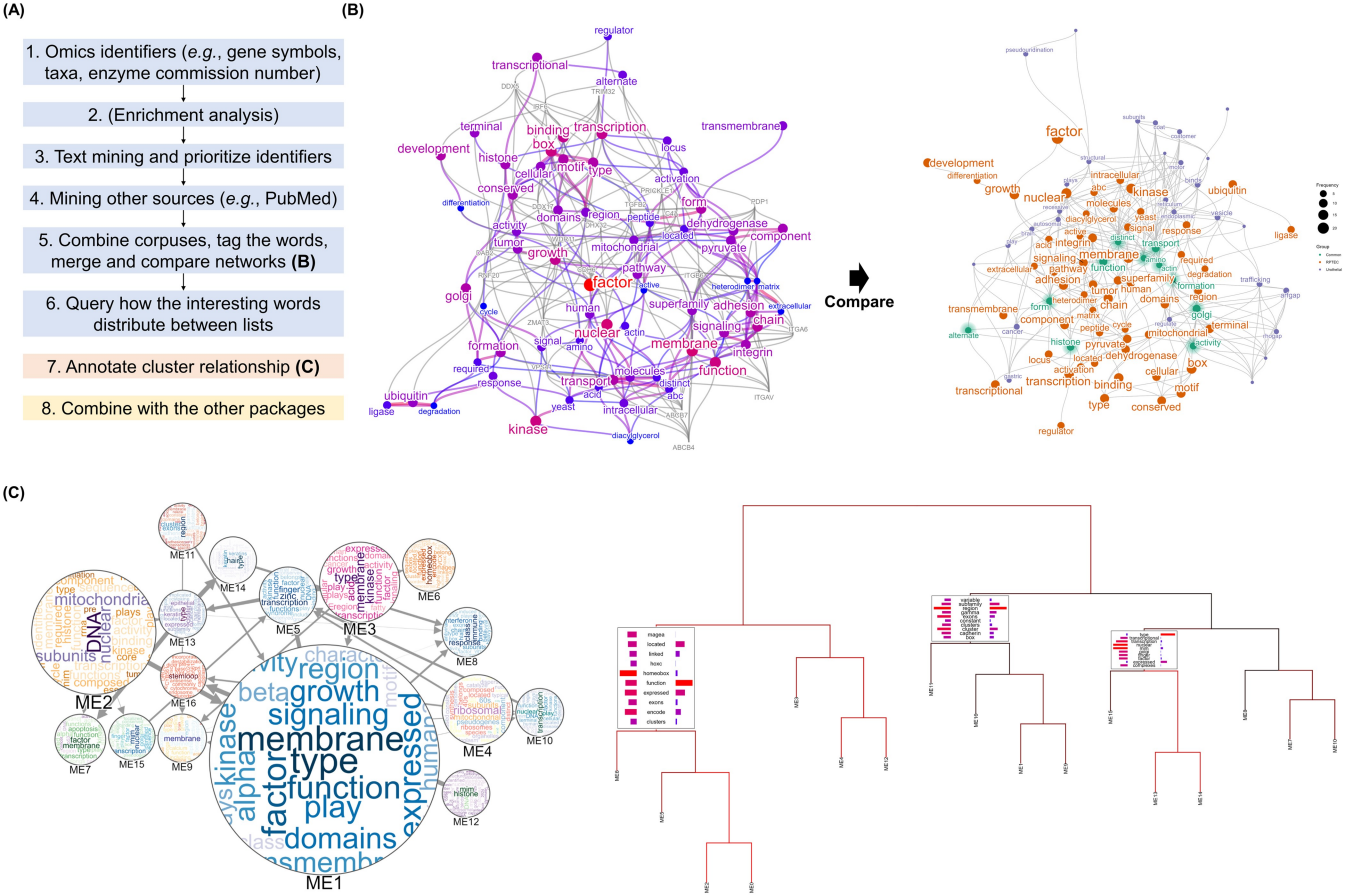
The example visualization using the *biotextgraph* package. (A) The overview of the workflow using the package. (B) The summarization using a correlation network obtained using the queries of interested gene identifiers (symbol) and RefSeq summary obtained by analyzing RNA-seq dataset of BKPyV infection in RPTEC, with the candidate genes related to high-frequent words. The result was compared to the other network derived from analyzing the other cells. (C) Bayesian network of gene clusters obtained from the RNA-seq dataset of bladder cancer was annotated using the package and exported to interactive visualization with Cytoscape.js. Wordclouds inside the nodes correspond to textual information in the cluster, and edge width corresponds to the strength of interaction. The dendrogram representing how the words are distributed across branches, which can be used not only for the gene identifiers but for microbial taxa.

Additional customization of networks can be achieved according to specific objectives. For instance, if there is an interest in sequential words, keyword analysis such as n-gram analysis can be conducted owing to the package *tm* or *quanteda* (Feinerer *et al.* 2008, Benoit *et al.* 2018). The anticipated workflow is shown in Fig. 1A.

### 2.2 Gene identifiers

For datasets involving transcriptomics or gene identifiers, the package fetches data from biomedical data resources including RefSeq summary (O’Leary *et al.* 2016), the gene description of Alliance of Genome Resources (Alliance of Genome Resources Consortium 2020), the description of EA results performed on gene lists from databases like Kyoto Encyclopedia of Genes and Genomes (KEGG) and Reactome (Kanehisa and Goto 2000, Wu *et al.* 2021), and PubMed information. The users can customize visualization through filtering by the preset Gene Ontology terms, for obtaining the biologically relevant information (Ashburner *et al.* 2000).

### 2.3 Microbial identifiers

The textual description of microbiome signatures can be also summarized. For this purpose, the package fetches textual information from curated microbial databases linking diseases to taxa, BugSigDB, and its R port *bugsigdbr* (Geistlinger *et al.* 2023), description of enzyme commission numbers (Gasteiger *et al.* 2003), user-defined microbiota-related metabolites (Dekkers *et al.* 2022), PubMed information, or the metabolic pathway description in the curated databases for metabolic functions across all domains, MetaCyc (Caspi *et al.* 2020).

The summarization and combination of textual information from these sources can be useful for characterizing a group of interesting taxa, their contributions to diseases and metabolic capabilities, as well as their similarity with other sets of taxa in terms of text, leading to the interpretation of possible functionality of microbiome and microbiome-derived genes.

### 2.4 Other visualization and analysis options

For the growing number of biomedical databases across domains, the manual function providing the same functionality as above is prepared. User-customized databases or text not listed in the package can be used for scalability. This enables the comparison and merging of different identifier lists using textual information.

The visualization function of the package includes not only the network but also those producing wordclouds and bar plots representing the metrics such as word frequencies. These produced plots can be reflected in various analyses and plots produced by the other packages related to omics analysis. In gene cluster analysis like weighted gene co-expression analysis (WGCNA) (Langfelder and Horvath 2008), it is often the case that we perform the analysis assessing the relationship between clusters. It is possible to annotate the networks between clusters using textual information. In the network, nodes represent clusters and edges indicate relationships between them. The results can be exported to interactive interface, Cytoscape.js and vis.js, which can be easily styled and modified including node sizes and edge widths, and can be inspected (Franz *et al.* 2015). The export to the interactive user interface is available for all the graphs produced in the package for the visualization of complex networks consisting of many variables.

The other options include the plots produced from workflows of single-cell transcriptomics analysis (Morabito *et al.* 2021), and the dendrogram plots representing the relationship between biological entities like bacterial taxa from the microbiome analysis. The usage for the package is in the documentation available on GitHub. For users not familiar with R language, the web application is available using Shiny (Chang *et al.* 2022).

## 3 Results

### 3.1 Application example in transcriptomic analysis

We presented an R package *biotextgraph* for text mining the various omics’ summary data. We first demonstrate the use of the package for analysis of the RNA-seq dataset investigating transcriptomic changes of renal proximal tubular epithelial cells (RPTEC) induced by BK polyomavirus (BKPyV) infection (Assetta *et al.* 2016). BKPyV is a DNA virus that infects multiple organs in patients with immunosuppressive treatments after solid organ transplantation. The aim of the analysis was to discover the functionality of these genes and the possible candidate genes related to infection not identified in differential expression analysis and EA. The differentially expressed mRNAs at 3 days post-infection are obtained using DESeq2 (Love *et al.* 2014). Using downregulated gene symbols not in the enriched biological pathways as input, the correlation network based on word frequency showing between-word relationships derived from RefSeq description as well as the associated gene symbols to highly frequent words were obtained. We found the possible downregulation of the transcriptional regulation, ubiquitin ligase degradation, and membrane-related transcripts which cannot be directly assessed by performing only EA or assessing log2 fold changes (Fig. 1B) (Caller Laura et al. 2019).

In addition to investigating a list of genes, it is often the case that the similarity of identified lists between multiple datasets is of interest. In this example, BKPyV is known to infect the bladder in addition to the kidney, thus we used an RNA-Seq dataset of BKPyV-infected normal urothelial cells for comparison with changes in RPTEC (Baker *et al.* 2022). The aim of the analysis was to assess the similarities and differences in cellular responses to infection. Similarly, downregulated genes were obtained using DESeq2, and there were no statistically significant enriched pathways using Reactome (Jassal *et al.* 2020). Using the functions from *biotextgraph*, the integrated networks of RefSeq summary data from two networks were visualized (Fig. 1B). The results suggested that trafficking through Golgi apparatus was involved and downregulated in both conditions assessed from textual information.

### 3.2 Application in investigating microbiome signature

The textual information in microbial signatures identified in differential abundance analysis can also be summarized. We obtained the taxa significantly enriched in Crohn's disease patients from BugSigDB. We query the genus name on MetaCyc pathway description to summarize how these taxa have metabolic functionality in the gut microbiome. Intersection of obtained taxa and described taxa in MetaCyc were taken, and the last taxonomic level of identified taxa in MetaCyc and its corresponding pathway’s description was used as the input for the manual function in the package. The produced plot is depicted in Supplementary Fig. S1. The results suggested that the package can successfully capture relationships such as Crohn's disease and sialic acids (Chen *et al.* 2022).

### 3.3 Application combined with the other packages

The biological network is often used to understand the complex relationships between biological entities, like gene regulatory networks. The package can be used to populate the network of clusters of biological entities for an intuitive understanding of cluster relationships.

We demonstrate here the example using a transcriptomic dataset investigating bladder cancer, GSE133624. We inferred Bayesian network of module eigengenes using WGCNA and *bnlearn* (Scutari 2010, Chen *et al.* 2019). We used the library to produce an interactive graph of BN between modules with contained text information using wordclouds (Fig. 1C). This enables the creation of plots that intuitively convey the size of gene clusters, their regulatory relationships, and functional implications as assessed by textual information.

The package can be used in conjunction with the other workflows or plots used in omics analysis. Annotation of the dendrogram of biological entities or clusters using textual information can be used to interpret how the elements are separated with regard to text frequency (Galili 2015). This can aid in inspecting the relationships between clusters or entities obtained from any analysis method in terms of words. The dendrogram inferred from the same dataset as the BN example above is shown in Fig. 1C. The annotation can be shown on the branches leading to user-specified tips. The EA results can also be plotted on the dendrogram for this purpose. The other examples include annotating marker genes of the clusters in single-cell transcriptomic analysis (Lun *et al.* 2016). The visualization results can be projected onto the other plots such as a dimensionality reduction plot, which helps understand the clustering results from the textual information (Supplementary Fig. S2).

## 4 Conclusion

We presented an R package *biotextgraph* with its usage in analyzing transcriptomic and microbiome datasets. We showed how the package can help interpret the lists of biological entities through network representation and biomedical text resources. In addition, the usage of how the package can be combined with the other workflows or packages in omics analysis is presented. The limitation of the package includes the lack of reproducibility when the web-based resources are used, as they are updated daily. The function includes the time-stamp for the produced object and offers the functionality for limiting the date range. Also, the long list of the entities cannot be handled by the web-based functions due to the data size limit. Trials for changing the arguments to obtain the informative graph for the input are sometimes necessary.

This package could aid in interpreting lists of biological entities from the available literature conveniently by measuring functional implications based on the network representation of their textual description. The package would contribute to hypothesis generation and knowledge discovery.

## Supplementary Material

btae357_Supplementary_Data

## Data Availability

The data underlying this article are available in the article and in its online supplementary material.
