# Supplementary material for: biotextgraph: graphical summarization of functional similarities from textual information
Source: Bioinformatics. 2024 Jun 8;40(6):btae357. doi: 10.1093/bioinformatics/btae357 (PMC11198732; doi:10.1093/bioinformatics/btae357)
Supplement: btae357_Supplementary_Data [file btae357_supplementary_data.docx]

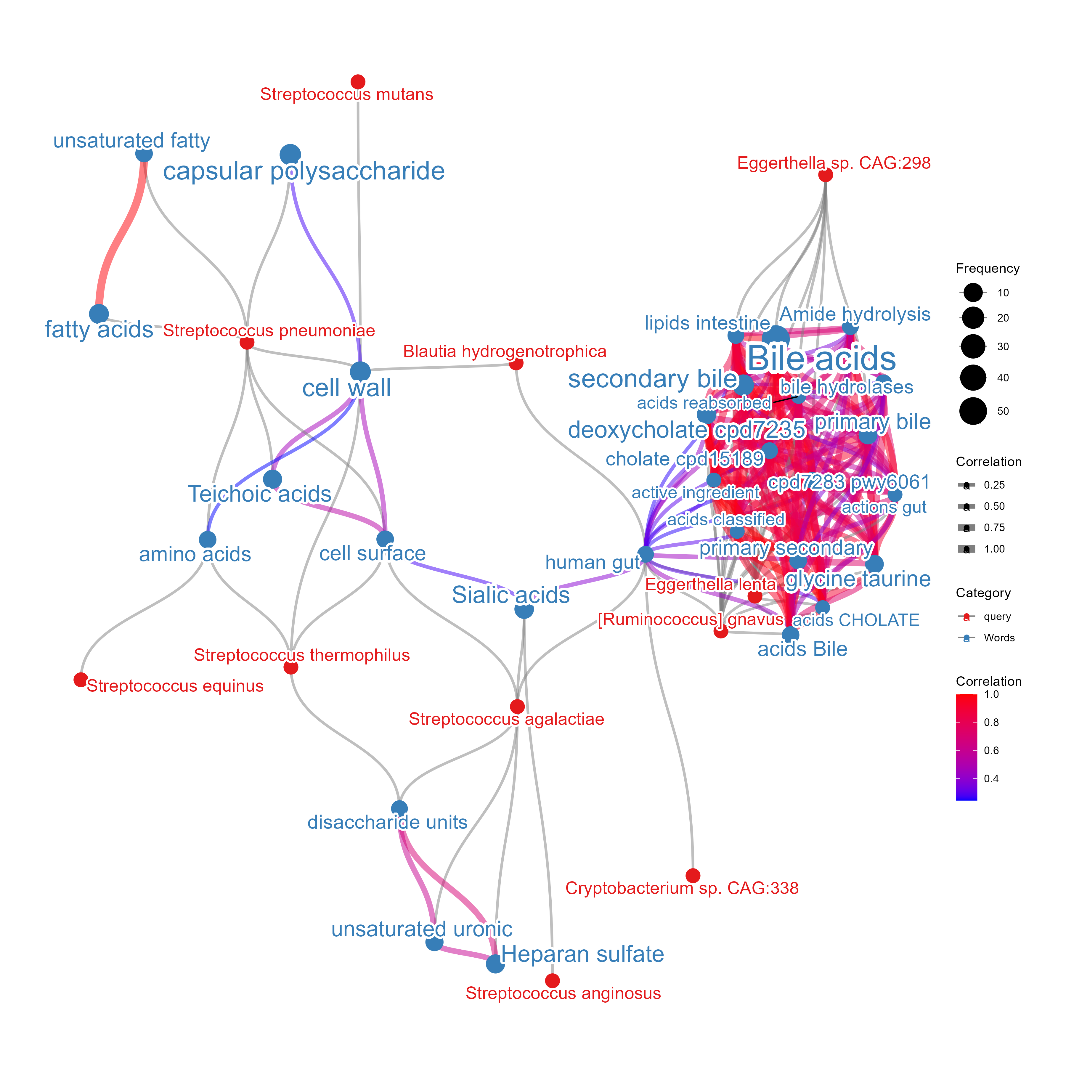


**Supplementary Figure 1. The summarization of MetaCyc pathway information regarding upregulated taxonomy in Crohn’s disease patients.** Using bugsigdbr, genus abundant in Crohn’s disease patients are obtained, and queried to obtain MetaCyc pathway description data. The resulting text is summarized by bi-gram tokenization as network visualization with corresponding species names.

**
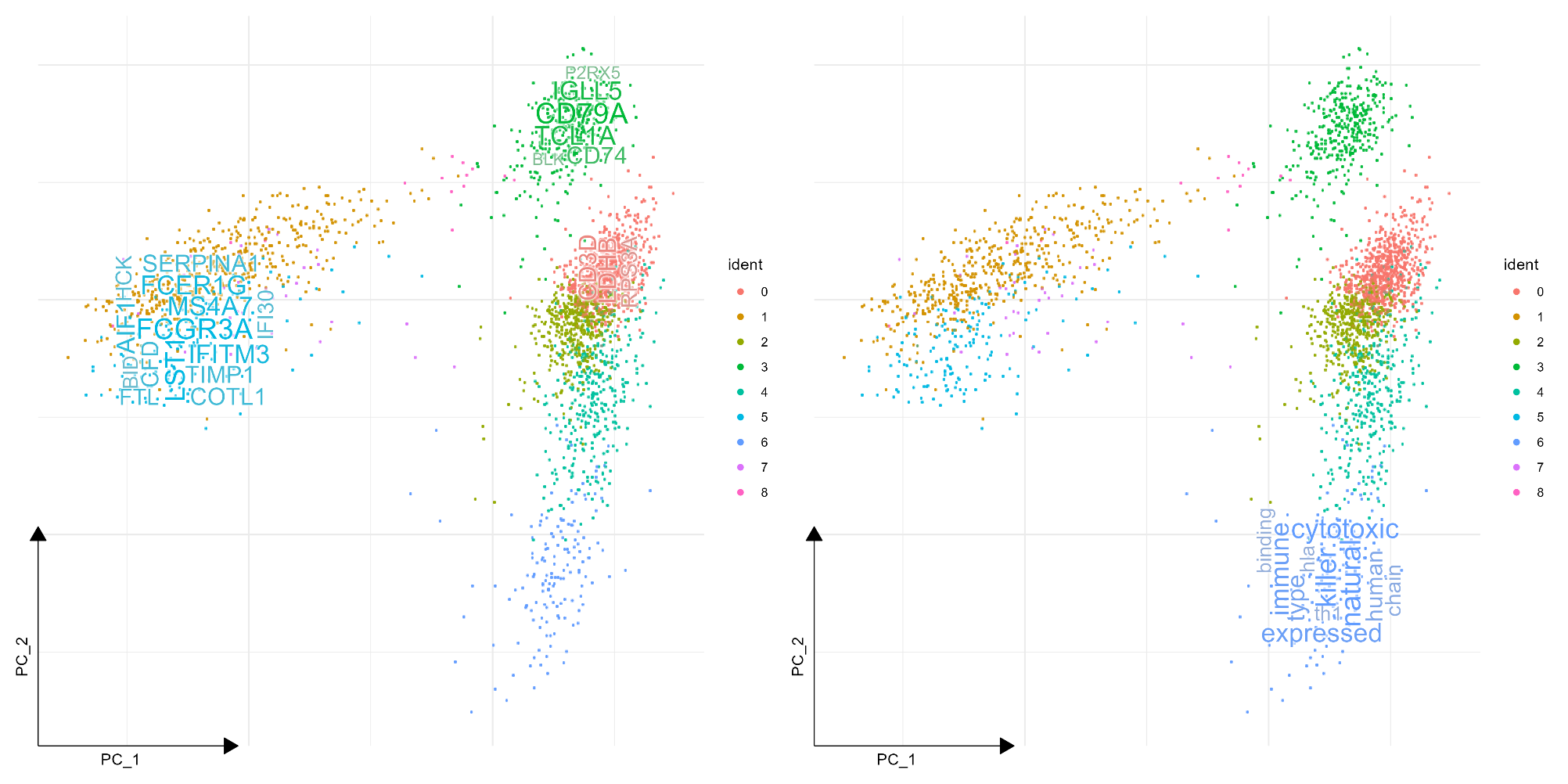
**

**Supplementary Figure 2. Annotation of single-cell transcriptomic data.** The reduced dimension plots were annotated by the presented library for the wordclouds of gene marker names. Principal component analysis was performed and the point color indicates the clusters. Gene descriptions can be also plotted and compared with the annotation results of cell clusters.
